# Intestinal permeability in human nonalcoholic fatty liver disease: A systematic review and meta‐analysis

**DOI:** 10.1111/liv.14696

**Published:** 2020-10-21

**Authors:** Toon J. I. De Munck, Pan Xu, Harm J. A. Verwijs, Ad A. M. Masclee, Daisy Jonkers, Jef Verbeek, Ger H. Koek

**Affiliations:** ^1^ Department of Internal Medicine Division of Gastroenterology and Hepatology Maastricht University Medical Centre+ Maastricht the Netherlands; ^2^ School of Nutrition and Translational Research in Metabolism (NUTRIM) Maastricht University Maastricht the Netherlands; ^3^ Department of Gastroenterology and Hepatology University Hospitals KU Leuven Leuven Belgium

**Keywords:** gut‐liver axis, intestinal barrier, intestinal permeability, nonalcoholic fatty liver disease, nonalcoholic steatohepatitis

## Abstract

**Background:**

The gut‐liver axis is considered to play a critical role in the development and progression of nonalcoholic fatty liver disease (NAFLD). The integrity of the epithelial barrier is crucial to protect the liver against the invasion of microbial products from the gut, although its exact role in NAFLD onset and progression is not clear.

**Methods:**

We performed a systematic review and meta‐analysis of studies that addressed the intestinal permeability (IP) in association with NAFLD presence or severity as defined by the presence of nonalcoholic steatohepatitis (NASH) and the degree of steatosis, hepatic inflammation or fibrosis. A total of 14 studies were eligible for inclusion.

**Results:**

Studies investigating IP in adult (n = 6) and paediatric (n = 8) NAFLD showed similar results. Thirteen of the included studies focussed on small IP, two studies on whole gut permeability and none on colonic permeability. In the pooled analysis, NAFLD patients showed an increased small intestinal permeability compared to healthy controls based on dual sugar tests (standardized mean difference 0.79, 95% CI 0.49‐1.08) and serum zonulin levels (standardized mean difference 1.04 ng/mL, 95% CI 0.40‐1.68). No clear difference in IP was observed between simple steatosis and NASH patients. Furthermore, whole gut and small intestinal permeability increased with the degree of hepatic steatosis in 4/4 studies, while no association with hepatic inflammation or fibrosis was observed.

**Conclusion:**

Based on the limited number of studies available, IP appears to be increased in NAFLD patients compared to healthy controls and is associated with the degree of hepatic steatosis.

**Abbreviations**
^51^Cr‐EDTA: ^51^Cr‐labelled ethylenediaminetetraacetic acid
BMI: body mass index
h: hour
HC: healthy control
IP: intestinal permeability
L/M: lactulose to mannitol ratio (L/M)
L/R: lactulose to rhamnose ratio
LPS: lipopolysaccharide
NAFLD: nonalcoholic fatty liver (simple steatosis)
NAFLD: Nonalcoholic fatty liver disease
NAS: NAFLD activity score
NASH: nonalcoholic steatohepatitis
PEG: polymers of polyethylene glycol
S/E: sucralose to erythritol ratio

**Highlights**

- To date, the role of intestinal permeability (IP) in human NAFLD is not clear.
- Studies investigating IP in NAFLD mostly focus on small intestinal permeability.
- IP appears to be increased in NAFLD patients and appears to be positively associated with the degree of hepatic steatosis.
- IP is not associated with the degree of hepatic inflammation or fibrosis.

## 1 INTRODUCTION

Nonalcoholic fatty liver disease (NAFLD) is the most common liver disease in the Western world in both adults and children. NAFLD prevalence is approximately 25% in the adult population and 8% in the paediatric population.^1^, ^2^ The spectrum of NAFLD ranges from nonalcoholic fatty liver (NAFL), nonalcoholic steatohepatitis (NASH) to liver fibrosis, cirrhosis and hepatocellular carcinoma.^1^ To date, the exact pathophysiology of NAFLD has not completely been elucidated and it is not clear to what extent paediatric NAFLD differs from adult NAFLD.^3^

The interaction between the gut and the liver, the so‐called ‘gut‐liver axis’, is considered to play a critical role in development and progression of NAFLD in both children and adults.^4^ Crosstalk between gut and liver is facilitated through the intestinal barrier. This intestinal barrier consists of structural elements (mucus and closely lined epithelial cells sealed by tight junctions), immune cells and soluble mediators (eg IgA, antimicrobial peptides).^4^ An intact intestinal barrier is able to restrict translocation of bacterial products, while allowing active transport from nutrients across the tight junctions.^4^ The epithelial integrity of the intestinal barrier can be assessed in vivo by measuring the intestinal permeability (IP). Increased IP can lead to translocation of microbial products from the gut to the liver through the portal system. Known factors that contribute to an increased IP include consumption of a Western diet (ie high fat intake), gut microbiome perturbations, pro‐inflammatory cytokines, alcohol and use of antibiotics.^5^, ^6^

Currently, a number of non‐invasive tests to measure IP in humans are being used. Urinary recovery of orally administered sugars (ie sucrose, lactulose to mannitol ratio (L/M), lactulose to rhamnose ratio (L/R), sucralose to erythritol ratio (S/E) and sucralose) are widely accepted as markers for IP. Five hour (h) urinary sucrose levels are used as indicator for gastroduodenal permeability, 5‐6 h L/M and L/R as indicators for small intestinal permeability, and 5‐24 h or 0‐24 h S/E as indicators for colon and whole gut permeability respectively. By using the ratio of two sugars with different size and therefore different transport mechanism (paracellularly versus transcellularly), correction for differences in renal function, intestinal transit time and gastric emptying is possible.^7^ Urinary recovery of a single substance cannot correct for these factors, which can differ between patients and thereby affect the outcome. Other substances used to measure IP in vivo are various polymers of polyethylene glycol (PEG) and ^51^Cr‐labelled ethylenediaminetetraacetic acid (^51^Cr‐EDTA).^7^ More recently, zonulin, a 47‐kDa protein, has been introduced as a potentially useful systemic marker for small intestinal and gastroduodenal permeability, but not for colon permeability.^8^ Serum zonulin has emerged as a relevant biomarker because it is an important factor to regulating IP by modulating intercellular tight junctions.^9^, ^10^ However, the specificity of serum zonulin as biomarker for small intestinal permeability remains uncertain.^11^

In both adults and paediatric NAFLD patients, several studies investigated IP and its role in the pathogenesis and progression from NAFL to NASH.^12^, ^13^, ^14^, ^15^, ^16^, ^17^ However, the exact association between IP and NAFLD severity (degree of steatosis, hepatic inflammation, fibrosis or presence of NASH) is not clear. The aim of this systematic review and meta‐analysis was to summarize studies in humans on the association between in vivo IP alterations and NAFLD presence and/or severity. We hypothesize that IP is increased in NAFLD, being most pronounced in progressive disease as characterized by the presence of NASH, advanced steatosis, hepatic inflammation or hepatic fibrosis. Furthermore, in the included studies, we will summarize the clinical parameters (eg anthropometric data and blood biochemical variables), which have been observed to correlate with the degree of IP in NAFLD patients.

## 2 METHODS

Reporting of this systematic review and meta‐analysis was performed according to the PRISMA guidelines (preferred reporting items for systematic reviews and meta‐analyses).^18^

### 2.1 Search strategy

A systematic literature search was conducted in 2020 (week 38) in both PubMed and Embase. The following keywords, synonyms and MeSH terms were used: non*alcoholic fatty liver disease, Nonalcoholic Fatty Liver Disease, fatty liver disease, NAFL, Fatty Liver, Non‐alcoholic Fatty Liver Disease, non*alcoholic steatohepatitis, NASH, nonalcoholic steatohepatitis, liver steatosis, hepatic steatosis, liver steatosis, intestinal barrier, gut barrier, gut permeability, permeability, zonulin. This resulted in 1070 hits and after exclusion of duplicates, 783 were included for screening of abstracts and full text. In addition, references of selected articles were assessed and included if suitable.

### 2.2 Eligibility criteria

Studies on in vivo IP measurements in human NAFLD patients were included in this systematic review. Studies were eligible for inclusion when the following inclusion criteria were met: (i) original peer reviewed research paper in English, (ii) the study population or a subgroup of the population consist of NAFLD patients (diagnosed with liver biopsy or imaging) without cirrhosis (because cirrhosis itself can lead to an increased IP), (iii) in vivo IP measurements (ie by urinary excretion of orally administered sugars, ^51^Cr‐EDTA or polyethylene glycol or by serum zonulin levels) and (iv) comparison of IP between groups (healthy controls (HC) vs NAFLD or NAFL vs NASH). Exclusion criteria included: (i) review articles, letter to the editor, commentaries, (ii) animal studies, (iii) studies investigating ex vivo permeability (ussing chambers) and solely microbial translocation via endotoxin/ lipopolysaccharide (LPS) levels.

### 2.3 Selection process and data extraction

To reduce selection bias, all titles and abstracts were screened for eligibility (based on in‐ and exclusion criteria) independently by two authors (HV, TDM). After consensus full text of selected articles were again independently checked for eligibility by the same authors (HV, TDM). Furthermore, both authors independently extracted all data using standardized data extraction forms. Data on patient characteristics (HC and NAFLD), method of NAFLD diagnosis (imaging or biopsy), IP test, main outcome (IP comparison between groups and relationship to liver histology) and observed correlations between the degree of IP and clinical factors was extracted. In case of disagreement on eligibility, the two reviewers came to consensus after discussing the article with a third reviewer.

### 2.4 Quality assessment

The methodological quality of the selected studies was assessed by two independent researchers (HV, TDM) using the Newcastle‐Ottawa Quality Assessment Scale (NOS) for case‐control studies.^19^ The NOS‐score was converted to (Agency for Healthcare Research and Quality) AHRQ standards using the following thresholds: good quality: 3 or 4 stars in the ‘selection’ domain AND 1 or 2 stars in ‘comparability domain’ AND 2 or 3 stars in ‘exposure’ domain. Fair quality: 2 stars in the ‘selection’ domain AND 1 or 2 stars in ‘comparability domain’ AND 2 or 3 stars in ‘exposure’ domain. Poor quality: 0 or 1 star in the ‘selection’ domain OR 0 stars in ‘comparability domain’ OR 0 or 1 stars in ‘exposure’ domain.

### 2.5 Statistical analysis

Meta‐analyses were performed using a random effect model with Review Manager version 5.3 if at least two studies evaluated a similar IP marker and compared this marker between HC and NAFLD patients or between biopsy proven NASH and NAFL (NAFLD not NASH) patients. Because of different test characteristics studies investigating small intestinal permeability by means of urinary recover of orally administered sugars (5‐6 h L/M or L/R) and by means of serum zonulin, were pooled separately. Because of differences in physicochemical properties, data on 24 h urinary collection of ^51^Cr‐EDTA and sucralose were not pooled. If both a BMI matched (or obese) control group and a normal weight control group were available in one study, data of the BMI matched control group was used in the analysis. All data were entered as mean ± SD. When the original results were only reported as median and (IQR) we estimated mean and SD using the formula proposed by Wan et al.^20^ In the studies where the data were included in figures and not provided numerically, we used software program Plot Digitizer to extract data. The pooled standardized median difference with 95% CIs were presented in forest plots. Heterogeneity of study results was tested with χ^2^ and *I* ^2^ calculations. We intended to assess publication bias by visual examination of the funnel plot and the Egger test for funnel plot asymmetry.

## 3 RESULTS

### 3.1 Study selection

**FIGURE 1:**
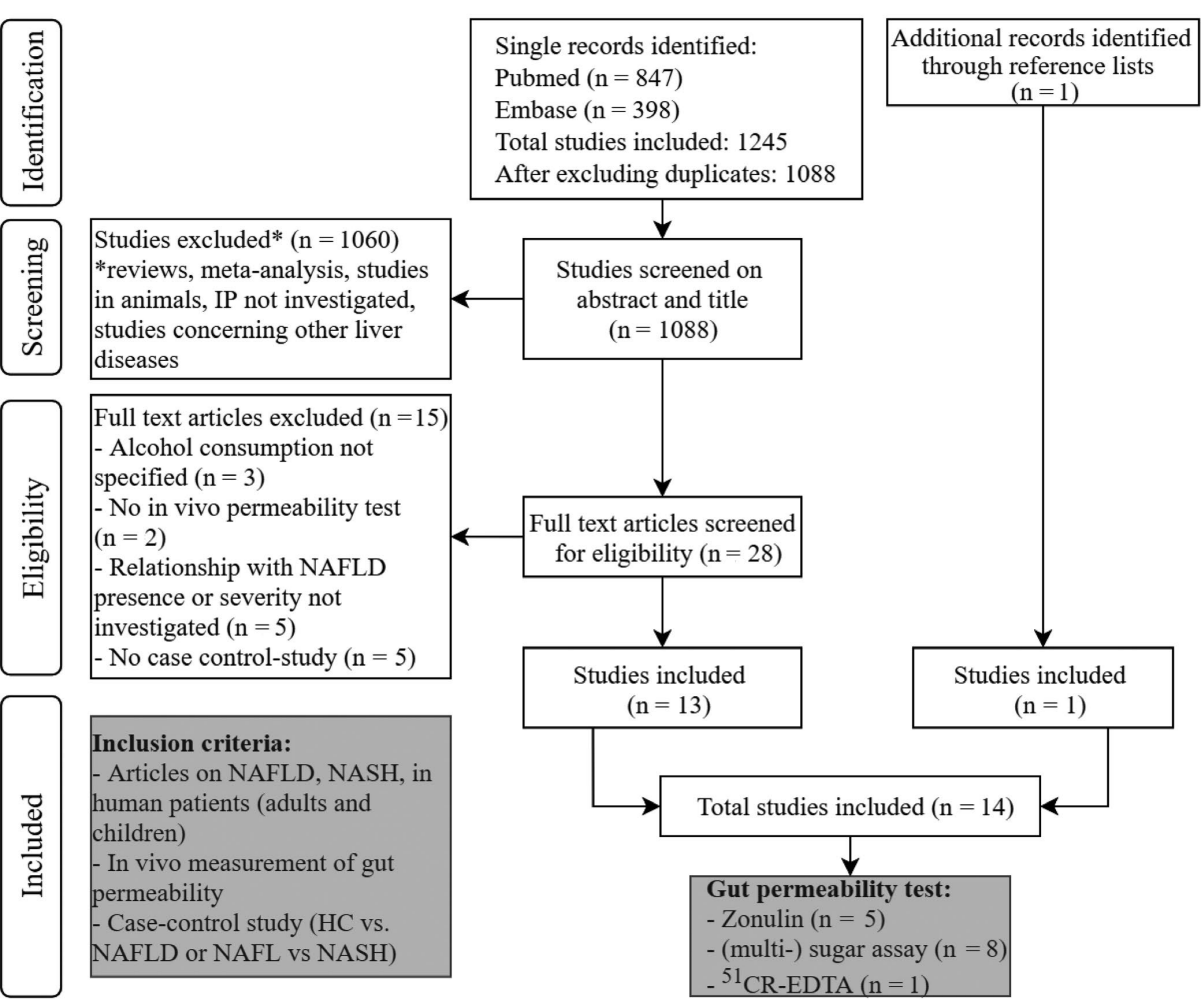
Flowchart of the selection process. NAFLD, nonalcoholic fatty liver disease; HC, healthy control; NAFL, nonalcoholic fatty liver (simple steatosis); NASH, nonalcoholic steatohepatitis

Twenty‐eight studies were eligible for full text screening. Thirteen of 28 studies matched the criteria and were included in this review. One additional study was identified through reference checking. Excluded studies did not specify alcohol consumption (n = 3), did not use an in vivo IP test (n = 2), did not investigate the association between IP and NAFLD presence or severity (n = 5) or did not include a control group (HC or NAFL) (n = 5). Further details on the selection process can be found in the flowchart (Figure 1).

### 3.2 Study characteristics

Nine studies investigated IP with urinary recovery of orally administered molecules (ie sugars or ^51^Cr‐EDTA) (Table 1) and five studies investigated IP with serum zonulin levels (Table 2). Only two studies investigated whole gut permeability by means of 24 h urinary recovery of ^51^Cr‐EDTA or sucralose while all other studies focused on small intestinal permeability.^12^, ^21^ In five of fourteen studies BMI was not significantly different between the control group and NAFLD group. However, in only two studies BMI matching of the control group with the NAFLD group (implemented in the study design) was performed.^14^, ^22^ Nine (5 adult and 4 pediatric) of fourteen studies used the golden standard, liver biopsy, to diagnose NAFLD, while the five other studies (one adult and four paediatric) used ultrasound. Study characteristics of all included studies are summarized in Table 1 (urinary recovery of orally administered molecules) and Table 2 (serum zonulin).

**TABLE 1:** Overview of studies investigating intestinal permeability using urinary recovery of orally administered probes

Author, country	Adult/children	NAFLD	Method NAFLD	HC	Method HC	IP test	Main outcome
Pierri et al 2018, Italy^36^	Children	5 NAFLD	US	5 HC (OB)	US	L/M (5h)	L/M = in NAFLD vs HC (*P* not given)
Troisi et al 2017, Italy^37^	Children	10 NAFLD	US	24 HC ‐ 10 HC^a^ (OB) ‐ 14 HC (*NW*)	US US	L/M (5h)	L/M ↑ in NAFLD vs HC (NW) (*P* < .003) L/M = in NAFLD vs HC^d^ (OB) (*P* > .003)
Guercio Nuzio et al 2017, Italy^24^	Children	11 NAFLD	US	21 HC ‐ 12 HC (OB) ‐ 9 HC (*NW*)	US US	L/M (5h)	L/M ↑ in NAFLD vs HC (NW) (*P* = .002) L/M ↑ in NAFLD vs HC^d^ (OB) (*P* = .002)
Nobili et al 2015, Italy^16^	Children	80 NAFLD ‐ 31 NAFL ‐ 49 NASH	Biopsy NAS NAS^b^	NP	/	L/M (6h)	L/M ↑ in NASH vs NAFL (*P* < .001)
Giorgio et al 2014, Italy^15^	Children	40 NAFLD ‐ 21 NAFL ‐ 18 NASH	Biopsy NAS NAS^b^	21 HC	US	L/M (6h)	L/M ↑ in NAFLD vs HC (*P* < .05) L/M ↑ in NASH vs NAFL (*P* < .05)
Volynets et al 2012, Germany^17^	Adult	20 NAFLD	US	10 HC	US	L/M (6h)	L/M ↑ in NAFLD vs HC (*P* < .05)
Miele et al 2009, Italy^12^	Adult	35 NAFLD ‐ 18 NAFL ‐ 17 NASH	Biopsy NAS 1‐4 NAS ≥ 5	24 HC^a^	US	^51^Cr‐EDTA (24h)	^51^Cr‐EDTA ↑ in NAFLD vs HC (*P* < .001)
Farhadi et al 2008, USA^21^	Adult	16 NAFLD ‐ 6 NAFL ‐ 10 NASH	Biopsy^c^	12 HC	BMI < 25	L/M (5h) + Sucralose (24h)	L/M = NAFL/NASH/HC (*P* not given) Sucralose = NAFL/NASH/HC (*P* not given)
Wigg et al 2001, Australia^38^	Adult	18 NASH	Biopsy^d^	16 HC	liver function	L/R (5h)	L/R NASH = IP HC (*P* = .37)

Abbreviations: NP, Not Present; NAFLD, nonalcoholic fatty liver disease; NASH, nonalcoholic steatohepatitis; NAFL, simple steatosis; HC, healthy control; OB, Obese; NW, Normal Weight; US, ultrasound; IP, intestinal permeability; NAS, NAFLD activity score; L/M, lactulose mannitol ratio; L/R, lactulose rhamnose ratio; ^51^Cr‐EDTA, chromium‐51 ethylenediaminetetraacetic acid excretion.

^a^ BMI not significantly different between HC and NAFLD.

^b^ Diagnosis based on NAS. All patients with NASH had minimal: steatosis (1‐3), lobular inflammation (1‐3) and ballooning (1‐2).

^c^ Some degree of hepatocellular steatosis, and characteristic lobular mixed inflammation is sufficient to diagnose NASH.

^d^ Steatosis and any inflammation is sufficient for the diagnosis of NASH.

**TABLE 2:** Overview of studies investigating intestinal permeability using serum zonulin

Author, Country	Adult Children	NAFLD	Method NAFLD	HC	Method HC	IP test	Main outcome
Loffredo et al 2017, Italy^39^	Children	67 NAFLD ‐ 44NAFL ‐ 23 NASH	Biopsy NAS 1‐4 NAS ≥ 5	72 HC	Not specified	Serum zonulin	‐ zonulin ↑ in NAFLD vs HC (*P* = .0001) ‐ Zonulin = in NASH vs NAFL (*P* = .10)
Cakir et al 2017, Turkey^40^	Children	28 NAFLD	US	30 HC	Not specified	Serum zonulin	Zonulin ↑ in NAFLD vs HC (*P* = .049)
Pacifico et al 2014, Italy^14^	Children	40 NAFLD ‐ 15 NAFL ‐ 25 NASH	Biopsy NAS NAS^b^	40 HC^a^	MRI	Serum zonulin	Zonulin ↑ in NAFLD vs HC (*P* = .009)
Hendy et al 2017, Egypt^22^	Adult	56 NAFLD ‐ 24 NAFL ‐ 32 NASH	Biopsy NAS 1‐4 NAS ≥ 5	20 HC^a^	Biopsy	Serum zonulin	‐ zonulin ↑ in NAFLD vs HC (*P* < .001) ‐ zonulin ↑ in NASH vs NAFL (*P* < .001)
Chwist et al 2014, Poland^13^	Adult	70 NAFLD ‐ 43 NAFL ‐ 27 NASH	Biopsy NAS 1‐4 NAS ≥ 5	NP	NP	Serum zonulin	zonulin ↑ in NASH vs NAFL (*P* = .003)

Abbreviations: NP, not present; NAFLD, nonalcoholic fatty liver disease; NASH, nonalcoholic steatohepatitis; NAFL, simple steatosis; HC, healthy control; US, ultrasound; IP, intestinal permeability; NAS, NAFLD activity score.

^a^ BMI not significantly different between HC vs NAFLD.

^b^ Diagnosis based on NAS. All patients with NASH had minimal: steatosis (1‐3), lobular inflammation (1‐3) and ballooning (1‐2).

### 3.3 Quality of included studies and risk of bias

Table S1 summarizes the quality of all included studies using the adapted NOS. Eight studies had poor quality. Most of these studies scored poorly on the comparability domain, with BMI matching of the control group in only two of the included studies.^14^, ^22^ Several factors within all included studies cause heterogeneity across studies. Five different IP tests are used within the 14 studies (zonulin, L/M (5‐6 h), L/R (5 h), sucralose (24 h) and ^51^Cr‐EDTA (24 h)). In total, eight studies (5 pediatric and 3 adult) investigated small IP by means of L/M (5‐6 h) (7 studies) or L/R (5 h) (1 study), of which data could be extracted and pooled in forest plots. Similarly, data of five studies (three paediatric and two adults) investigating small IP by means of serum zonulin could be pooled. Data of two studies investigated whole gut permeability by means of 24 h urinary collection of sucralose and ^51^Cr‐EDTA were not be pooled because of heterogeneity. Publication bias was not assessed as there were inadequate numbers of included trials in each analysis (less than 10) to properly assess funnel plot asymmetry.^23^

### 3.4 Small intestinal permeability in NAFLD versus HC

**FIGURE 2:**
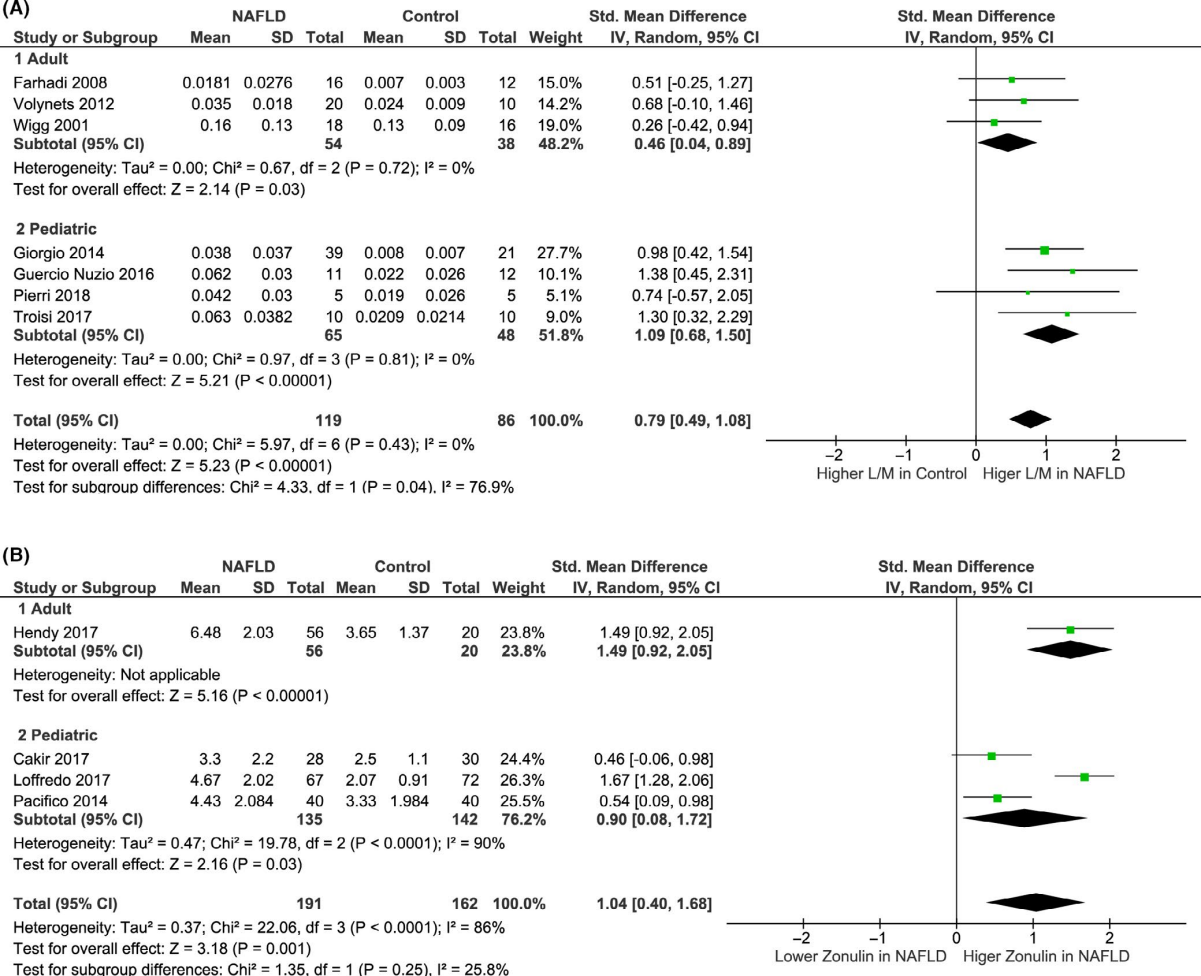
Forest plots of small intestinal permeability in NAFLD patients vs healthy controls. (A) Studies using L/M (lactulose to mannitol ratio) or L/R (lactulose to rhamnose ratio) to measure IP; (B) studies using serum zonulin (ng/mL) to measure IP. Including subgroup analysis by age (adult vs paediatric)

Figure 1A shows the quantitative synthesis of the mean L/M or L/R levels of NAFLD subjects vs HC. Seven studies (three adult and four paediatric) comprising a total of 119 NAFLD patients (54 adult and 65 paediatric) and 86 HC (38 adult and 48 paediatric) were included. Overall, NAFLD patients showed an increased small intestinal permeability by means of L/M or L/R (standardized mean difference 0.79 95% CI 0.49‐1.08 compared to HC (Figure 2A). The statistical heterogeneity between studies was low (*I* ^2^ = 0%). Small intestinal permeability by means of L/M or L/R was increased in both adult and paediatric NAFLD patients compared to HC. However, in the subgroup analysis (adult vs paediatric), the paediatric population showed a significantly higher difference in L/M between the study groups (standardized mean difference 1.09, 95% CI 0.68‐1.50 compared to HC).

Four studies (1 adult and 3 pediatric) comprising a total of 191 NAFLD patients (135 paediatric and 56 adult) and 162 HC (142 pediatric and 20 adult) were included in the quantitative synthesis of the mean serum zonulin levels in NAFLD patients and HC (Figure 1B). Overall, NAFLD patients had an increased small intestinal permeability by means of serum zonulin (standardized mean difference 1.04 ng/mL, 95% CI 0.40‐1.68 as compared to HC (Figure 2B). The statistical heterogeneity between studies was high (*I* ^2^ = 86%). In the subgroup analysis (Figure 2B), serum zonulin levels were observed to be increased in both adult and paediatric NAFLD patients compared to HC.

### 3.5 Small intestinal permeability in NASH vs NAFL

**FIGURE 3:**
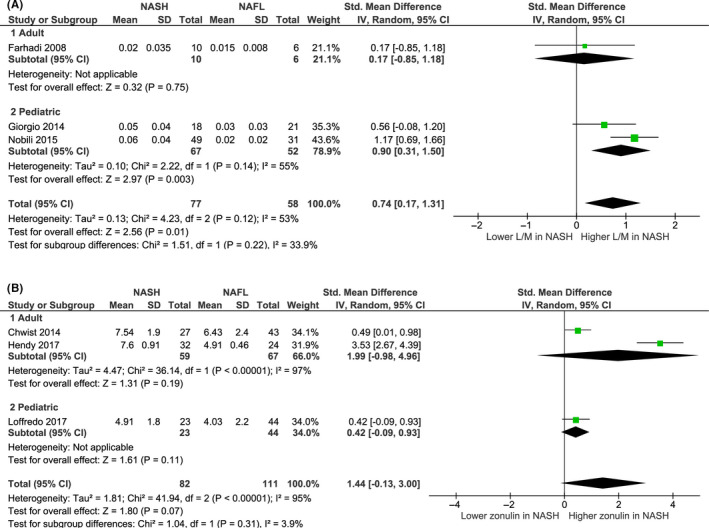
Forest plots of small intestinal permeability in NASH vs NAFL patients. (A) Studies using L/M (lactulose to mannitol ratio) to measure IP, (B) studies using serum zonulin (ng/mL) to measure IP. Including subgroup analysis by age (adult vs paediatric)

Three studies (one adult and two paediatric) comprising 77 NASH patients (67 paediatric and 10 adult) and 58 NAFL patients (52 paediatric and 6 adult) were included in the quantitative synthesis of the mean L/M levels of NASH vs NAFL patients (Figure 2A). Overall NASH patients had an increased small intestinal permeability by means of L/M (standardized mean difference 0.74, 95% CI 0.17‐1.13) compared to NAFL patients (Figure 3A). The statistical heterogeneity between studies was substantial (*I* ^2^ = 53%). In the subgroup analysis (Figure 3A), mean L/M was significantly increased in paediatric NASH patients compared to paediatric NAFL patients while this was not the case for adult patients (Figure 3B).

Three studies (two adult and one paediatric) comprising 82 NASH patients (23 paediatric and 59 adult) and 111 NAFL patients (44 paediatric and 67 adult) were included in the quantitative synthesis of the mean serum zonulin levels of NASH vs NAFL patients (Figure 2B). Overall NASH patients had no significantly different serum zonulin levels compared to NAFL patients (standardized mean difference 1.44 ng/mL, 95% CI −0.13‐3.00, *I* ^2^ = 95%) (Figure 3B). When pooled separately (Figure 3B), in both adult and paediatric patients, no difference in serum zonulin levels between NASH and NAFLD patients was observed.

### 3.6 Whole gut permeability in NAFLD

Two adult studies investigated whole gut permeability in adult NAFLD patients by means of 24 h urinary collection of ^51^CR‐EDTA or sucralose.^12^, ^21^ Data were not pooled because different markers were used. In the study of Farhadi et al, 24 h sucralose excretion was not significantly different between HC (n = 12), NAFL (n = 6) and NASH (n = 10) patients (Table 1).^21^ In the study of Miele et al 24 h ^51^CR‐EDTA excretion was significantly increased in NAFLD patients (n = 35) compared to HC (n = 24) and an increased ^51^CR‐EDTA excretion (median split) was not associated with the presence of NASH.^12^

### 3.7 Association between small intestinal permeability and NAFLD severity

Five of the included studies investigated the association between small intestinal permeability and one or more parameters of NAFLD severity (degree of steatosis, fibrosis, ballooning or inflammation) (Table 3). The association between small intestinal permeability and the degree of hepatic steatosis was investigated in three studies (two paediatric and one adult) (Table 3).^14^, ^15^, ^16^ In all studies more advanced hepatic steatosis was associated with an increased small intestinal permeability. To quantify hepatic steatosis all studies used the histological NAFLD activity score (NAS) (Tables 1 and 2). The association between small intestinal permeability and hepatic fibrosis was investigated in four studies (three pediatric and one adult),^13^, ^14^, ^15^, ^16^ while three paediatric studies investigated the association with hepatic inflammation and hepatic ballooning (Table 3).^14^, ^15^, ^16^ Only in the study of Giorgio et al, a positive correlation between L/M and the degree of portal inflammation, ballooning and fibrosis was observed in 12 paediatric NASH patients with increased L/M.^15^ In all other studies no association between small intestinal permeability and hepatic fibrosis, inflammation or ballooning was observed.

**TABLE 3:** Investigated association between IP and NAFLD severity

Author	IP Test	Type analysis	Group	Association between IP and NAFLD severity variables
Miele et al	^51^CR‐EDTA (24 h)	Median split ^51^CR‐EDTA ≤ 4.88% (n = 17) vs ^51^CR‐EDTA > 4.88% (n = 18) Mann‐Withney	35 NAFLD	The degree of lobular inflammation, hepatic ballooning, hepatic fibrosis or number of NASH diagnosis was *not significantly different* between groups.
		S1 (n = 11) vsS2‐3 (n = 24) Mann‐Withney	35 NAFLD	^51^Cr‐EDTA ↑ in S2‐S3 vs S1 (*P* < .001)
Nobili et al	L/M (6 h)	Univariable linear regression	80 NAFLD	‐ Hepatic steatosis (β = 0.229, *P* = .042). ‐ No correlation with: (*P‐value NP*) Lobular inflammation hepatic ballooning hepatic fibrosis NAS
Giorgio et al	L/M (6 h)	Spearman's correlation	12 NASH (L/M ≥ 0.03)	Pathological L/M had a positive correlation with the degree of portal inflammation (*P* = .02), ballooning (*P* = .003) and fibrosis (*P* = .0002) (*r not given*)
		L/M < 0.03 (n = 27) vs L/M ≥ 0.03 (n = 12) Chi‐squared	39 NAFLD	S2‐3 prevalence ↑ in L/M ≥ 0.03 vs L/M < 0.03 (*P* = .0008)
Hendy et al	Serum Zonulin	Pearson correlation	56 NAFLD	NAS score (*r* = .518, *P* < .001)
Pacifico et al	Serum Zonulin	Spearman's correlation	40 NAFLD	‐ Hepatic steatosis (*r* = .372, *P* < .05) ‐ No correlation with: lobular inflammation (*P* = .23), ballooning (*P* = .10), fibrosis (*P* = .18), presence of NASH (*P* = .17).
		Multiple linear regression adjusted for BMI, abdominal fat, WBISI	40 NAFLD	MRI Hepatic fat fraction (β = 0.415, *P* < .05).
Chwist et al	Serum Zonulin	F0‐1 (n = 54) vs F2‐3 (n = 16) *t* test	70 NAFLD	Serum zonulin F0‐1 = F2‐3 (*P* = .67)

Abbreviations: NAFLD, nonalcoholic fatty liver disease; NASH, nonalcoholic steatohepatitis; NAS, NAFLD activity score; L/M, lactulose mannitol ratio; ^51^Cr‐EDTA, chromium‐51 ethylenediaminetetraacetic acid excretion; NP, not present; S0‐3, histological scoring system for hepatic steatosis; F0‐4, histological scoring system for hepatic fibrosis.

### 3.8 Association between whole gut permeability and NAFLD severity

The association between whole gut permeability (24 h ^51^Cr‐EDTA) and NAFLD severity was investigated in one adult study. Miele et al observed that 24 h ^51^Cr‐EDTA excretion was significantly increased in NAFLD patients with moderate to severe steatosis (S2‐3) compared to NAFLD patients with minimal or mild steatosis (S1).^12^ Furthermore, no difference in degree of hepatic fibrosis, hepatic inflammation or ballooning was observed between patient with normal and increased 24 h ^51^Cr‐EDTA excretion.^12^

### 3.9 Factors that significantly correlated with IP in NAFLD patients

Four of the included studies (one adult and three paediatric) reported significant correlations between small intestinal permeability and clinical factors including anthropometric data and blood biochemical variables (Table 4). Two studies observed a positive correlation between small intestinal permeability and the degree of insulin resistance.^14^, ^22^ In addition two other studies observed a positive correlation between small intestinal permeability and systemic LPS levels.^16^, ^24^ Other correlations ie with BMI, systolic blood pressure and blood ALT, IL‐6, triglycerides, γ‐GT and HDL‐C levels are only observed in single studies (Table 4).

**TABLE 4:** Investigated correlations with IP in NAFLD patients

Author	IP Test	Type analysis	Group	Association between IP and clinical variable
Hendy et al	Serum Zonulin	Pearson correlation	56 NAFLD	BMI (*r* = .378), ALT (*r* = .312), triglycerides (*r* = .296), HDL‐C (*r* = −.397), HOMA‐IR (*r* = .413), serum IL‐6 (*r* = .288).
Pacifico et al	Serum Zonulin	linear regression adjusted for age, gender and pubertal status	Total group ‐ 40 HC ‐ 40 NAFLD	‐ γ‐GT (β = 0.229), 2 h insulin (β = 0.340), WIBSI (β = −0.236)
Guercio Nuzio et al	L/M (5 h)	Pearson correlation	Total group ‐ 11 NAFLD ‐ 21 HC	Serum LPS (*r* = .48)
Nobili et al	L/M (5h)	Univariable linear regression	80 NAFLD	Systolic blood pressure (β = 0.196), Plasma LPS (β = 0.296).

Abbreviations: L/M, lactulose mannitol ratio; NAFLD, nonalcoholic fatty liver disease; HC, healthy control, BMI, body mass index; HDL‐C, high density lipoprotein cholesterol; ALT, alanine transaminase; HOMA‐IR, homeostasis model of assessment‐insulin resistance; γ‐GTP, gamma‐glutamyl transpeptidase; WIBSI, whole body insulin sensitivity; LPS, lipopolysaccharide.

## 4 DISCUSSION

In this systematic review, the association between in vivo IP and NAFLD and its severity was evaluated, based on eight studies in paediatric and six in adult NAFLD patients. In this study, we demonstrated that NAFLD patients have an increased IP compared to HC. Furthermore, we observed a positive correlation between IP and the degree of hepatic steatosis, while no clear association between IP and the presence of NASH, hepatic inflammation or fibrosis was demonstrated.

In the present systematic review, we included both studies in paediatric and in adults NALFD patients. Although paediatric NAFLD shows some different characteristic as compared to adults patients, such as histological features and progression rate to hepatic cirrhosis or HCC, they also show large overlap as both are associated with the metabolic syndrome, central obesity, dysregulated glucose metabolism, dyslipidemia, cardiovascular diseases and have similar genetic risk factors (eg PNPLA3 and GCKR).^25^ In addition, gut microbiome dysbiosis, metabolic endotoxemia and systemic inflammation are considered to play a role in paediatric and adult NAFLD development.^26^ To account for possible differences between adult and paediatric patients, subgroup analysis by age was performed and discussed below.

First, we observed that NAFLD patients had increased small intestinal permeability by means of L/M, L/R (seven studies) or serum zonulin levels (four studies) compared to HC. IP was also found to be increased in NAFLD compared to controls when only considering studies that matched for BMI or where BMI did not differ between groups. This indicates that the presence of NAFLD, independent of BMI, is associated with an increased IP. Furthermore, we observed a more prominent increase in small intestinal permeability by means of L/M in paediatric NAFLD patients compared to adult NAFLD patients. IP in NAFLD patients is believed to be influenced by, among others, microbiome perturbations, faecal short chain fatty (SCFA) acids levels and endogenous alcohol production.^27^ Since microbiome composition is different in children compared to adults, altered production of SCFAs and alcohol by the intestinal microbiota and difference in IP are expected. In line, butyrate and propionate were observed to be enriched in faecal samples from adult NAFLD patients, while formate, acetate and valerate, were less abundant whereas butyrate and propionate were unaffected in faecal samples from paediatric NAFLD patients.^28^, ^29^ As IP was often investigated in a singular paediatric or adult study, subgroup analysis by age is not desirable. Therefore, future studies are needed to investigate differences in IP between adult and paediactric NAFLD patients.

Evidence is less convincing when comparing NAFL and NASH patients as investigated in six studies. In the pooled analysis, an increased small intestinal permeability was found by L/M (three studies) but not zonulin (three studies). It should be noted that results need to be interpreted with care because of substantial heterogeneity between studies for both parameters. Furthermore the number of study subjects in both adults or paediatric studies is very low. In previous studies, IP has also been associated with several metabolic abnormalities including obesity, dyslipidaemia and hyperglycaemia.^5^ In NAFLD patients, increased IP is believed to induce hepatic steatosis, inflammation and fibrosis via translocation of bacterial products from the gut to the liver.^4^

Furthermore, we investigated the association between IP and NAFLD severity. We demonstrated that small intestinal permeability increases with the degree of hepatic steatosis while no association with hepatic inflammation, ballooning or fibrosis was observed in the included studies. Interestingly, it cannot be excluded that an increased IP is more important in the development of hepatic steatosis than of hepatic inflammation or fibrosis. Based on experimental and cross‐sectional data, not only an association of increased IP with the degree of hepatic steatosis but also with hepatic inflammation and fibrosis was expected. The development of hepatic fibrosis and inflammation is believed to be triggered by bacterial translocation from the gut to the liver, however, a causal link has not been proven.^4^, ^6^, ^28^ Mechanistically, translocation of bacterial products (eg LPS), leads to activation of toll‐like receptor 4 in the liver and results in hepatic inflammation and fibrogenesis.^4^, ^6^, ^28^ The amount of NAFLD subjects with significant hepatic fibrosis or inflammation in the included studies is relatively low what may explain why no association between IP and hepatic fibrosis or inflammation was found. Furthermore, most studies included in this review focus on small intestinal permeability, while colon permeability was not investigated. Microbiome perturbation in the colon have been associated with NAFLD presence and severity and are believed to harm the integrity of the gut barrier.^28^, ^30^ In mice, high fat diet feeding has been observed to induce metabolic abnormalities, systemic and liver inflammation which was accompanied by an increased colon permeability.^31^ Furthermore, Pijls et al observed an increased colon permeability in patients with stable compensated cirrhosis compared to healthy controls while gastroduodenal and small IP were not altered.^32^ Possibly colon permeability is linked to the degree of hepatic inflammation and fibrosis in NAFLD patients while small intestinal permeability is not. Therefore, future studies should also focus on the association between colon permeability and hepatic fibrosis and inflammation.

We identified only two human studies evaluating whole gut permeability in NAFLD patients. ^12^, ^21^ In the study of Miele et al whole gut permeability was increased in adult NAFLD patients compared to HC and was associated with more advance steatosis but not with hepatic inflammation, ballooning or fibrosis.^12^ In the study of Farhadi et al no association between whole gut permeability and NAFLD presence or severity was observed.^21^ More research is needed to elucidate the role of whole gut permeability in NAFLD patients.

Finally, we wanted to elucidate clinical factors that correlated with IP in NAFLD. However, data on this topic are scarce. Associations between small intestinal permeability and serum liver function tests, pro‐inflammatory cytokines, and metabolic factors are underinvestigated in the NAFLD population. In the general population, elevated levels of pro‐inflammatory markers, dyslipidaemia, hyperglycaemia, insulin resistance, anthropometric measurements resembling obesity and the consumption of a Western‐style diet have been identified as confounding factors for IP.^5^ These factors may vary between different chronic diseases and because of the limited data confounding factors for increased IP in NAFLD patients remain to be identified.

When comparing study results, IP test characteristics must be taken in mind. Urinary recovery of enteral administrated non‐digestible markers (different sugars or ^51^Cr‐EDTA) are widely used to assess IP at different sites of the gastrointestinal tract (depending on type of marker and collection time). Duals sugar tests (eg L/M and L/R) have greater clinical value than the administration of one marker alone (sucralose or ^51^Cr‐EDTA) as they are less influenced by differences in renal function, intestinal transit time or gastric emptying time between study subjects. Recently serum zonulin has emerged as a marker to assess the small intestinal epithelial integrity. However, Ohlsson et al suggest that serum zonulin might rather be a biomarker for low‐grade inflammation than for IP, because zonulin is identical to prehaptoglobin‐2, not enterocyte specific and associated with overweight, obesity and hyperlipidemia.^10^, ^11^, ^33^, ^34^ Furthermore, in the study of Linsalata et al serum zonulin did not correlate with the L/M but did correlate with serum IL‐6 and serum IL‐8 concentrations in 91 subjects (39 irritable bowel syndrome, 32 coeliac disease and 20 HC). Finally, to date zonulin is the only known regulator of intestinal tight junction but it is likely that other zonulin unrelated pathways are also important in this process. Caution must be taken when using serum zonulin as a biomarker for small IP. Therefore, studies using zonulin as marker for small intestinal IP were analysed separately.^11^

This systematic review has some limitations. Firstly, because of the observational nature of all included studies in this systematic review only associations and not causalities were investigated. Secondly, substantial inter‐study heterogeneity was noted in most analyses. In this review, only studies investigating in vivo IP by means of urinary excretion of orally administered substances or serum zonulin levels were included. Studies using circulating LPS levels, the major component of the outer membrane of Gram‐negative bacteria, as marker for IP were not included as circulating LPS measurements are not site‐specific and have a high false‐positive rate.^35^ Thirdly, because of the small number of studies included in the meta‐analysis the presence of publication bias cannot be ruled out and subgroup analysis is not desirable. Finally, only 14 studies were included, which were small in terms of sample size, focused on both paediatric and adult NAFLD and most of them had poor quality.

In conclusion, small intestinal permeability appears to be increased in NAFLD patients compared to healthy controls and appears to be positively associated with the degree of hepatic steatosis. However, included studies where small in sample size, had poor quality and showed high heterogeneity. To date, no clear evidence is available that small intestinal or whole gut permeability increases with NAFLD severity (presence of NASH, hepatic inflammation or fibrosis). Future studies should also focus on colonic permeability in NAFLD patients.

## CONFLICTS OF INTEREST

All authors report no conflicts of interest relevant to this article.

## Supporting information

Supplementary Material
