# Supplementary material for: Intestinal permeability in human nonalcoholic fatty liver disease: A systematic review and meta‐analysis
Source: Liver Int. 2020 Oct 21;40(12):2906–16. doi: 10.1111/liv.14696 (PMC7756870; doi:10.1111/liv.14696)
Supplement: Supplementary file 1 — Supplementary Material [file LIV-40-2906-s001.docx]

Intestinal permeability in human nonalcoholic fatty liver disease: a systematic review and meta-analysis

Toon. J. I. De Munck^1,2^, Pan Xu^2^, Harm J. A. Verwijs^2^, Ad A. M. Masclee^1,2^, Daisy Jonkers^1,2^, Jef Verbeek^3^, Ger. H. Koek^1,2^

Search strategy ...........................................................................................................2

Table S1.......................................................................................................................3

**1. Search strategy**

PUPMED

(((((((((((((((((non*alcoholic fatty liver disease) OR "Nonalcoholic Fatty Liver Disease") OR "fatty liver disease")OR nafl*) OR "Fatty Liver"[Mesh]) OR "Non-alcoholic Fatty Liver Disease"[Mesh]))) OR ((non*alcoholic steatohepatitis) OR NASH) OR nonalcoholic steatohepatitis))))) OR liver steatosis[MeSH Terms]) OR hepatic steatosis) OR liver steatosis)) AND (((((((intestinal barrier) OR gut barrier) OR gut permeability) OR intestinal permeability) OR "Permeability"[Mesh])) OR zonulin)​

Single records identified: 847 (week 38 2020)

EMBASE

((fatty liver or nonalcoholic fatty liver or non*alcoholic fatty liver disease or NAFL*) or (non*alcoholic steatohepatitis or nonalcoholic steatohepatitis or NASH)) AND (((((intestinal barrier) OR gut barrier) OR gut permeability) OR intestinal permeability) OR zonulin)​

Filter: human

Single records identified: 398 (week 38 2020)

Total after exclusion duplicates: 1088

**2. Supplementary Table 1: Newcastle Ottawa Scale for assessment of quality of included case-control studies.**

|  | Selection | | | | Comparability | | Exposure | | |  |
| --- | --- | --- | --- | --- | --- | --- | --- | --- | --- | --- |
| First author, year | Adequate case definition | Representativeness of the cases | Selection of Controls | Definition of controls | Study controls matched for BMI | Study controls matched for age/sex | Ascertainment of exposure | Same method of ascertainment | Non-Response rate | **Total out of 9** |
| *Loffredo* (2019)^36^ | + | + | − | − | − | + | + | + | + | 6 |
| *Pierri (2018)^33^* | + | + | − | + | − | − | + | + | + | 6 |
| *Cakir* (2017)^37^ | + | + | − | − | − | − | + | + | + | 5 |
| *Troisi* (2017)^34^ | + | + | + | + | − | − | + | + | + | 7 |
| *Hendy* (2017)^22^ | + | + | + | + | + | + | + | + | + | 9 |
| *Guerico Nuzio* (2017)^23^ | + | + | − | + | − | − | + | + | + | 6 |
| *Nobili* (2015)*^16^ | + | + | − | + | − | − | + | + | + | 6 |
| *Pacifico* (2014)^14^ | + | + | − | + | + | + | + | + | + | 8 |
| *Chwist* (2014)*^13^ | + | + | − | + | − | − | + | + | + | 6 |
| *Giorgio* (2014)^15^ | + | + | − | + | − | + | + | + | + | 7 |
| *Volynets* (2012)^17^ | + | − | − | + | − | − | + | + | + | 5 |
| *Miele* (2009)^12^ | + | + | + | + | − | + | + | + | + | 8 |
| *Farhadi* (2008)^21^ | + | − | − | + | − | − | + | + | + | 5 |
| *Wigg* (2001)^35^ | + | + | − | + | − | + | + | + | − | 6 |

** NAFL vs NASH*

Legend: + = star, - = no star. green: good quality; yellow: fair quality; red: poor quality.
